# COX-2 Protects against Atherosclerosis Independently of Local Vascular Prostacyclin: Identification of COX-2 Associated Pathways Implicate Rgl1 and Lymphocyte Networks

**DOI:** 10.1371/journal.pone.0098165

**Published:** 2014-06-02

**Authors:** Nicholas S. Kirkby, Martina H. Lundberg, William R. Wright, Timothy D. Warner, Mark J. Paul-Clark, Jane A. Mitchell

**Affiliations:** 1 National Heart & Lung Institute, Imperial College London, London, United Kingdom; 2 The William Harvey Research Institute, Barts & the London School of Medicine & Dentistry, Queen Mary University of London, London, United Kingdom; McMaster University, Canada

## Abstract

Cyxlo-oxygenase (COX)-2 inhibitors, including traditional nonsteroidal anti-inflammatory drugs (NSAIDs) are associated with increased cardiovascular side effects, including myocardial infarction. We and others have shown that COX-1 and not COX-2 drives vascular prostacyclin in the healthy cardiovascular system, re-opening the question of how COX-2 might regulate cardiovascular health. In diseased, atherosclerotic vessels, the relative contribution of COX-2 to prostacyclin formation is not clear. Here we have used apoE^−/−^/COX-2^−/−^ mice to show that, whilst COX-2 profoundly limits atherosclerosis, this protection is independent of local prostacyclin release. These data further illustrate the need to look for new explanations, targets and pathways to define the COX/NSAID/cardiovascular risk axis. Gene expression profiles in tissues from apoE^−/−^/COX-2^−/−^ mice showed increased lymphocyte pathways that were validated by showing increased T-lymphocytes in plaques and elevated plasma Th1-type cytokines. In addition, we identified a novel target gene, rgl1, whose expression was strongly reduced by COX-2 deletion across all examined tissues. This study is the first to demonstrate that COX-2 protects vessels against atherosclerotic lesions independently of local vascular prostacyclin and uses systems biology approaches to identify new mechanisms relevant to development of next generation NSAIDs.

## Introduction

Cyclo-oxygenase (COX) is expressed in two isoforms: constitutive COX-1 and ‘inducible’ COX-2 [1]. COX-1 is associated with homeostatic processes including protection of the gastro-intestinal tract and thrombosis. COX-2 is induced by cytokines and mitogens, and contributes to inflammation, pain, angiogenesis and cancer. As such, COX-2 is the therapeutic target for non-steroidal anti-inflammatory drugs (NSAIDs) including diclofenac (Voltaren), and COX-2 selective NSAIDs including celecoxib (Celebrex) and rofecoxib (Vioxx). Drug development in this area, including study of the potentially promising chemopreventative benefits of COX-2 inhibitors in cancer, was dramatically halted in 2005 with the world-wide withdrawal of rofecoxib and the continued controversy surrounding the cardiovascular safety issues of these drugs.

In line with the protective roles of COX isoforms in the gut, traditional NSAIDs, which inhibit both isoforms of COX, are associated with serious and potentially fatal gastro-intestinal side effects [2], [3]. We now know that gastro-intestinal side effects are a result of the combined inhibition of COX-1 and COX-2 in the gastric mucosa [4] and, as such, highly selective COX-2 NSAIDs, such as rofecoxib spare the gut [5]. Despite this proven advantage, rofecoxib was voluntarily withdrawn by its manufacturers when results of a placebo controlled clinical trial (the APPROVe study) suggested an increased risk of atherothrombotic events [6]. We now know that, in general, all drugs that inhibit COX-2, including traditional NSAIDs, which also inhibit COX-1, are associated with increased risk of cardiovascular events. This particularly manifests as an increase in rates of myocardial infarction [1], [7], [8], [9], [10], which is consistent with data from animal models showing that COX-2 deletion or inhibition can produce a pro-thrombotic phenotype [11], [12], [13]. Similarly, some studies show a pro-atherogenic phenotype of COX-2 inhibition [14], [15] or deletion [13], [16], although other pharmacological studies have suggested no change [17], [18], [19], [20] or reduced atherosclerosis [21] with pharmacological blockage of COX-2. Taken together, we have no unifying explanation for the association between COX-2 inhibitor use and cardiovascular events in man.

Inhibition of urinary prostacyclin metabolites by drugs such as rofecoxib [22] and celecxoib [22] has been used as the main evidence to support the idea that COX-2 drives prostacyclin locally within the vessel wall [8], [23]. Accordingly, inhibition of COX-2 derived prostacyclin release by NSAIDs has been suggested to remove a local protective block on platelet reactivity, vascular inflammation and atherosclerosis [8]. However, our group [24], [25], and others [26], [27], [28], have demonstrated that it is COX-1 and not COX-2 that drives vascular prostacyclin production in a healthy cardiovascular system and that urinary metabolites do not reflect levels in the circulation [24], [29]. We have gone on to use an unbiased systems analysis of COX-2 gene expression and found that, whilst COX-2 is not in large vessels, it is expressed in discrete hot spots including within the thymus, kidney and brain [30]; all locations previously shown to be sites of COX-2 activity. These findings leave us with no clear explanation of how COX-2 inhibitors might influence cardiovascular health [24], especially in those without overt cardiovascular disease such as those participating in the APPROVe study [6] or in healthy mice [11].

Data from animal models and human tissue demonstrate that COX-2 can be induced by inflammation in atherosclerotic vessels [18], [31], [32], [33], [34]. In these conditions, therefore, COX-2 may provide an additional pathway for local production of protective prostacyclin. Atherosclerotic disease is likely to be prevalent in NSAID target populations, such as arthritis patients, and subclinical atherosclerosis is present in even apparently healthy individuals. As such, it is essential that the role of COX-2 in prostanoid generation in atherosclerotic vessels be understood in order for us to move the field of COX-2 biology and NSAID development forward. Previous studies have provided mixed results as to how total prostacyclin formation is altered in animal models of atherosclerosis [35], [36], [37] but to our knowledge only one has addressed the role of particular COX isoforms in this process. Using a cholesterol-fed rabbit model, Wong e*t al.* [38] demonstrated aortic prostacyclin and PGE_2_ formation to be unaltered by selective COX-2 inhibitors, but partially inhibited by a non-selective COX-1/COX-2 inhibitor. Although this was a model of mild fatty streak formation with only modest COX-2 induction and data using pharmacological tools must be interpreted cautiously, these results suggest that COX-1 remains the dominant isoform for prostacyclin generation in mildly atherosclerotic vessels. By contrast, recently published data from Tang *et al.* [39] show endothelial-specific deletion of COX-2 exacerbates atherosclerosis in an LDL receptor knockout model, suggesting a protective role for vascular COX-2-derived prostacyclin.

Here we have used a fat-fed apoE^−/−^ mouse model to investigate the effect of genetic deletion of COX-2 on local prostacyclin release in profoundly atherosclerotic vessels. We have gone on to establish the extent to which global COX-2 protects against atherosclerosis and used microarray and pathway analysis techniques to identify COX-2 regulated gene networks, which may provide new targets for understanding how COX-2 regulates cardiovascular health. Whilst these experiments do not yet deliver definitive answers, this study is the first to demonstrate that COX-2 protects the cardiovascular system independently of vascular prostacyclin and shows that systems biology approaches are valid in this area for the identification of new targets for NSAID-induced cardiovascular risk. This will, in turn, allow for the development of safe, efficacious, next generation NSAIDs.

## Methods

### Animals and induction of atherosclerosis

ApoE^−/−^/COX-2^−/−^ double knockout mice were generated by cross of COX-2^−/−^ mice [40] with apoE^−/−^ mice (C57Bl/6J background; Charles River, UK) for 2 generations to produce apoE^−/−^/COX-2^+/−^ mice, which were then inter-crossed. The resulting apoE^−/−^/COX-2^−/−^ progeny were identified by genomic PCR, with apoE^−/−^/COX-2^+/+^ littermates serving as controls. To accelerate atherosclerosis in these animals, 12 week old male and female apoE^−/−^/COX-2^−/−^ and apoE^−/−^/COX-2^+/+^ animals were fed a high fat, high cholesterol diet (21% fat, 1.25% cholesterol, cholate-free; D12108; Research Diets, USA) for 10 weeks. The reported effects of COX-2 deletion on atherosclerosis and vascular prostanoid release were apparent in both male and female mice.

At the termination of all studies, animals were killed by CO_2_ narcosis, and the vasculature immediately perfused with phosphate buffered saline (PBS) across the heart. For some studies, mice were additional perfused with 10% neutral buffered formalin at physiological pressure.

This study was carried out in strict accordance with the guidelines set out under Animals (Scientific Procedures) Act 1986. All procedures have been approved by Imperial College Ethical Review Panel and under a designated Home Office License (PPL 70/7013) and were carried out by Home Office approved personnel. All efforts were made to minimise suffering.

### Optical projection tomography

Perfusion fixed aortic arches from apoE^−/−^/COX-2^+/+^ and apoE^−/−^/COX-2^−/−^ mice were cleaned of peri-adventitial material and embedded in agarose (Invitrogen, UK) before dehydration in methanol and optical clearing in 66% benzyl alcohol, 34% benzyl benzoate (Acros Organics, UK). Tissue morphology and plaque distribution was recorded by optical projection tomographic imaging of auto-fluorescent emission (405–445 nm excitation) using a Bioptonics 3001 tomograph as previously described [41]. Briefly, 400 projection images were recorded at 0.9° increments of rotation, and the three-dimensional volume reconstructed by Hamming-filtered back-projection (NRecon; Skyscan, Belgium). Plaque volume was quantified using CTan software (Skyscan, Belgium) by a blinded observer.

### Histological analysis and immunohistochemistry

Brachiocephalic arteries were collected from perfusion fixed apoE^−/−^/COX-2^+/+^ and apoE^−/−^/COX-2^−/−^ mice and post-fixed in 10% neutral buffered formalin (Sigma, UK) for 48 hrs, before dehydration and processing to paraffin wax. 5 µm thick serial sections were cut perpendicular to the vessel axis, and stained using Verhoeff's van Gieson method (Elastic Stain Kit; Sigma, UK). From each vessel, three representative sections were selected with at least 200 µm spacing. From photomicrographs of these, planimetric measurements of lesion size (defined as the area delineated by the luminal border and internal elastic lamina) were recorded using Photoshop CS5 Extended software (Abobe Systems Inc, USA). Lesion lipid content was estimated by measuring de-lipidated intra-plaque voids, as previously described [42].

Immunohistochemistry for α-smooth muscle actin (αSMA), Mac2, B220 and CD3 was performed according to the labeled streptavidin-biotin method using rehydrated paraffin sections. Before staining for B220, heat induced epitope retrieval was performed using pH 10 Tris-HCl antigen retrieval solution (Sigma, UK). For all stains, non-specific binding was blocked with 10% normal goat serum (Vector Labs, UK), 2.5% bovine serum albumin (Sigma, UK) and 2.5% non-fat milk powder (Marvel, UK) in PBS (Sigma, UK) Unconjugated primary antibodies were applied and incubated as indicated- anti-αSMA (Sigma, UK): 1/400 dilution; 30 min incubation; anti-Mac2 (Cederlane Labs, USA): 1/6000 dilution, overnight incubation; anti-B220 (Biolegend, UK): 1/200 dilution, overnight incubation; anti-CD3 (AbD Serotec, UK): 1/200 dilution, overnight incubation. Biotinylated goat anti-mouse IgG (αSMA) or goat anti-rat IgG secondary antibodies (Mac2, B220, CD3) were applied at 1/400 dilution for 30 mins, followed by streptavidin-conjugated horseradish peroxidase (Extravidin-Peroxidase; Sigma, UK; 1/400 dilution; 30 mins). Immunoperoxidase complexes were visualised by the application of 3,3-diaminobenzidine (Vector Labs, UK) and its development into an insoluble product. Specificity of staining was confirmed using appropriate positive and negative control tissues, and by substituting the primary antibody for an isotype matched irrelavent antibody. αSMA and Mac2 immunoreactivity, were quantified by colour deconvolution (Photoshop CS5 Extended, Abode Systems, USA) and the immunoreactive area expressed as a fraction of the total lesion area. B220 and CD3 were quantified by manual count by a blinded observer of immunoreactive cells in each of lesion, media and adventitia. Counts were expressed according to the total area of the appropriate vessel wall layer, measured as above.

### Sudan IV lipid staining

The aortic tree, including its major branches, was dissected from apoE^−/−^/COX-2^+/+^ and apoE^−/−^/COX-2^−/−^ mice perfused with PBS. Vessels were cut open to reveal their luminal surface, and pinned en face onto Sylgard coated plates (Dow Corning, USA). After brief fixation (10 mins; 10% neutral buffered formalin; Sigma, UK), vessels were rinsed in 70% ethanol before incubation (20 mins, room temperature) with filtered sudan IV solution (0.5% in 50% ethanol, 50% acetone; Sigma, UK). The stain was differentiated in 80% ethanol (5 mins), and vessels rinsed in running water. Aortas were photographed and the sudan IV-stained area quantified by image analysis (Photoshop CS5 Extended; Adobe Systems, USA) by a blinded observer.

### En face confocal imaging of the aortic arch

Perfusion fixed aortic arches were carefully dissected of peri-adventitial material and 2 mm rings of the proximal (between aortic root and base of the brachiocephalic artery) and mid-arch (between the base of the brachiocephalic artery and left carotid artery) removed for immunostaining. Briefly, aortic arch rings were treated with 0.1% Triton X-100 (Sigma, UK) and 20% normal goat serum (Vector Labs, UK; 12 hrs, room temperature) to permeabilize membranes and block non-specific binding, then incubated with primary antibodies raised against COX-1 (1∶50; 4 hrs, room temperature; Cayman Chemical, USA) or COX-2 (1∶50; 4 hrs, room temperature; Cayman Chemical, USA). Aortic rings were then treated with AlexaFluor-594-conjugated goat anti-rabbit IgG secondary antibodies (Invitrogen, UK) before incubation with Alexa-Fluor-488-conjugated anti-CD31 (1∶100; 12 hrs, 4°C; Biolegend, UK) and DAPI (25 µg/ml; 5min room temperature; Invitrogen, UK) to label endothelial cells and cell nuclei, respectively. Tissues were washed thoroughly with PBS between incubations. After staining, aortic rings were cut open to reveal the luminal surface, mounted flat between a glass slide and coverslip with aqueous hard-set media (Vector Labs, UK) and pressed until the media had firmly set.

The luminal surface of the arch was visualized with a Leica SP5 inverted confocal microscope at 40X objective magnification. Laser and gain settings were fixed at the beginning of each imaging protocol. Areas corresponding to lesser and greater curvature of the aortic arch were determined by tissue orientation and confirmed by the cell morphology in the CD31+ endothelial cell layer. In these regions, Z-stacks were recorded which included the endothelial cell, intra-plaque and medial layers. These layers were identified based cell and nuclear morphology and orientation, and CD31 immunoreactivity. Images presented and analysed represent a single plane from each Z-stack which best corresponds to the layer of vessel under study (i.e. endothelial layer, intraplaque layer or medial layer). Due to the uneven morphology of the atherosclerotic vessel, in some cases individual Z-stack planes may have cut across more than one vessel layer. COX-1 and COX-2 was quantified as mean fluorescence intensity using ImageJ software (National Institutes of Health, USA).

### Aortic COX activity bioassays

Aortic arch, brachiocephalic artery and thoracic aorta (regions both with and without grossly visible plaque formation) were isolated from animals perfused with PBS. Vessels were carefully dissected into 2 mm rings and placed immediately into individual wells of a 96 well microtitre plate containing DMEM (Sigma, UK) containing Ca^2+^ ionophore A23187 (50 µM; Sigma, UK). Vessels were incubated at 37°C for 30 mins then conditioned media was removed and subsequently analysed by competitive immunoassay for 6-keto-PGF1α (a stable breakdown product of prostacyclin; Cayman Chemical, USA) and PGE_2_ (Cisbio, France).

### qPCR

Tissues (heart, lung, liver, spleen, thymus, kidney) were removed from fat-fed apoE^−/−^ COX-2^+/+^ and apoE^−/−^ COX-2^−/−^ mice and snap frozen, homogenised, and total RNA extracted using a silica column-based kit (Nucleospin RNA II; Machery-Nagal, UK). RNA was converted to cDNA by reverse transcriptase (Superscript II; Invitrogen, UK) with oligo(dT) primers (Invitrogen, UK) and qPCR performed using a Rotor-Gene Q instrument (Qiagen, UK). Ptgs2 (probe ID: Mm00477214_m1), ptgs2 (probe ID: Mm00478374_m1) and rgl1 (probe ID: Mm00444088_m1) gene expression levels were determined using TaqMan expression assays and quantified relative to levels of 18S rRNA (probe ID: Mm03928990_g1) using the comparative Ct method.

### Microarray analysis

Total RNA extracted from apoE^−/−^/COX-2^+/+^ and apoE^−/−^/COX-2^−/−^ mouse lung, liver and thymus homogenates as above were subject to standard microarray procedures. Samples were converted to cDNA, fragmented, labelled, and hybridized to Illumina MouseRef-8v3 BeadChip arrays (Illumina, UK). Quality control and basic interpretation of data was performed at Bart's and The London Genome Centre, Queen Mary, University of London before datasets were received for further analysis. Data were subsequently analysed for differential expression levels and gene pathways using the Ingenuity iReport service (Ingenuity Systems, USA) and statistical significance determined using the linear models for microarray data (LIMMA) modified t-test method. Full methodological details can be found at http://www.ingenuity.com/getireport/pdf/ingenuity_robust_unattended_microarray_analysis.

### Blood cell and cytokine analyses

Venous blood was collected from the inferior vena cava. Plasma was separated from heparinized blood (10 U/ml; Leo Laboratories, UK) and levels of IL-1β, IL-2, IL-4, IL-10, IL-12 (total), IFNγ, KC and TNFα were measured using a multiplex immunoassay system (Meso Scale Diagnostics, USA). Plasma cholesterol and triglyceride levels and whole blood leucocyte counts were determined by a commercial veterinary diagnostics service (IDEXX Laboratories, UK).

### Statistics and data analysis

Data were analysed using Prism 5.0 software (Graph pad Software, USA) and are presented as mean±SEM. Data were compared by Student's unpaired t-test unless otherwise stated. ‘n’ refers to the number animals from which observations were made; where multiple measures were obtained from the same animal, values were averaged and considered as n = 1. Differences were considered significant if p<0.05.

## Results and Discussion

### Effect of COX-2 deletion on atherosclerosis in mice

**Figure 1:**
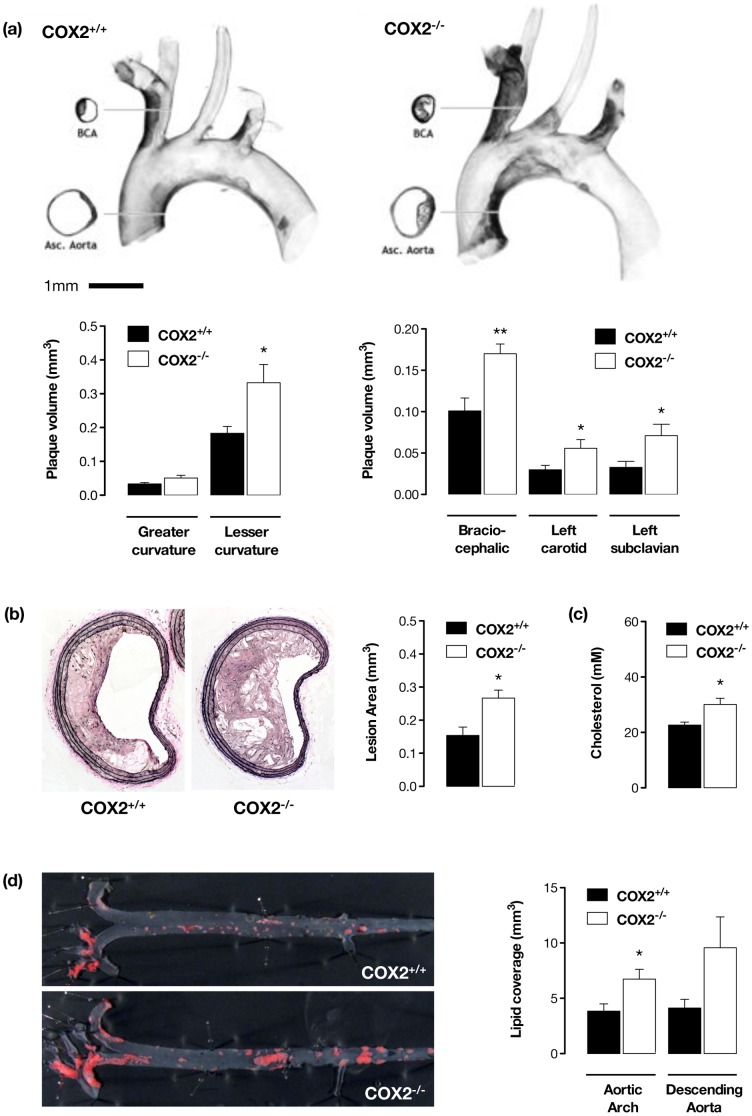
COX-2 deletion augments atherosclerosis in apoE ^−**/**−^ **mice.** Optical projection tomographic imaging of the aortic arch from fat-fed apoE^−/−^/COX-2^+/+^ and apoE^−/−^/COX-2^−/−^ mice (a) demonstrated a clear increase in atherosclerotic lesion burden in the lesser curvature of the arch, and its major branches: the brachiocephalic (BCA), left carotid and left subclavian arteries. Planimetric measurement of plaque area in van Gieson-stained histological sections of the brachiocephalic artery confirmed these observations (b), showing increased plaque area in apoE^−/−^/COX-2^−/−^ mice. Similarly, plasma cholesterol levels (c) and aortic lipid accumulation measured by *en face* sudan IV staining (d), were increased in apoE^−/−^ mice lacking COX-2. *; p<0.05 vs apoE^−/−^/COX-2^+/+^ by unpaired t-test; n = 8-12.

In man, NSAIDs, including COX-2 selective inhibitors, are associated with increased atherothrombotic events [10]. This suggests that COX-2 may protect the cardiovascular system against atherosclerosis. Experimental results in this area are not entirely united with increases [14], [15], decreases [21] and no change [17], [18], [19], [20] in atherosclerosis being reported in previous studies of pharmacological COX-2 inhibition. This possibly reflects differences in timing, model and the degree/selectivity of COX-2 inhibition achieved since any concurrent COX-1 inhibition is likely to be dominated by effects on platelet thromboxane and thereby protective against atherosclerotic lesion formation [18]. Importantly, in mice, both pre- [16] and post-natal [13] global COX-2 gene deletion is associated with increased atherosclerosis. In agreement, here we show that apoE^−/−^ mice developed severe atherosclerosis when fed a high fat, high cholesterol diet (Figure 1) and that this was exacerbated by deletion of COX-2. Atherosclerotic plaque burden (Figure 1a,b) and vascular lipid accumulation (Figure 1d) were all increased in apoE^−/−^/COX-2^−/−^ mice compared to littermate apoE^−/−^/COX-2^+/+^ control animals. This augmented atherosclerosis was associated with increased vascular inflammation, as indicated by an increase in accumulation of Mac2+ macrophage-like cells in the plaques, without alteration in plaque lipid or smooth muscle content (Figure S1). We also observed increased plasma cholesterol levels in apoE^−/−^/COX-2^−/−^ mice (Figure 1c) possibly reflecting previously described changes in lipoprotein function [16] and leucocyte-lipoprotein exchange pathways [43]. These increases in total plasma cholesterol alone however may not account for increased atherosclerotic burden since previous studies in this model show similar and greater increases in circulating cholesterol without increased plaque formation [44], [45].

### Role of COX-2 enzyme activity on prostacyclin production by atherosclerotic vessels

**Figure 2:**
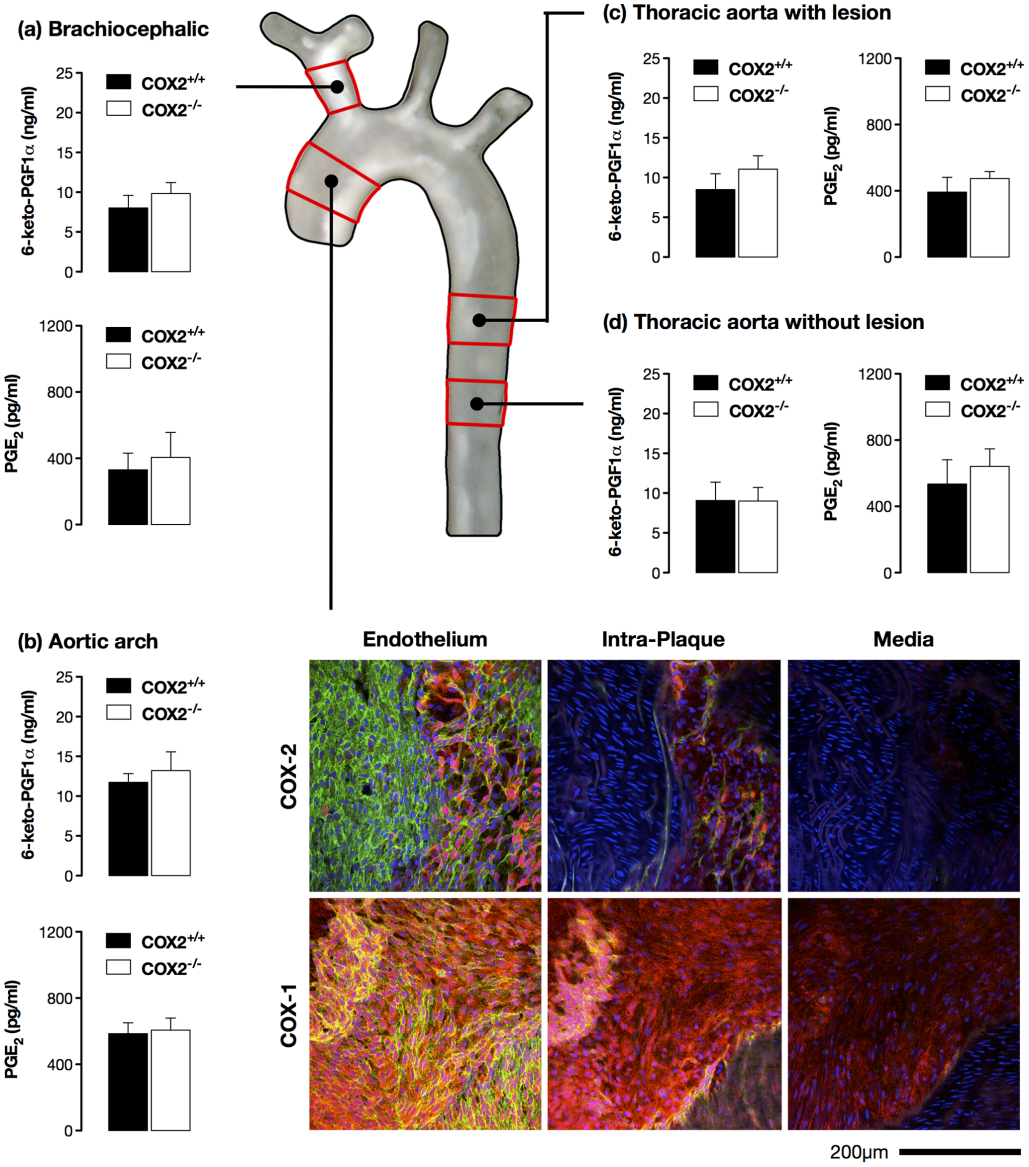
COX-2 deletion does not alter prostacyclin production by atherosclerotic vessels. Production of Prostacyclin and PGE_2_ by isolated rings of brachiocephalic artery (a) and aortic arch (b), and from plaque-bearing (c) and plaque-free regions of thoracic aorta (d) from fat-fed apoE^−/−^/COX-2^+/+^ and apoE^−/−^/COX-2^−/−^ mice demonstrated little role for COX-2 in the production of either prostaglandin in atherosclerotic vessels. In agreement, *en face* confocal immunofluorescence imaging of the lesser curvature of the aortic arch (b) suggests that whilst both COX-1 and COX-2-like immunoreactivity was present in atherosclerotic vessels, the COX-1 isoform was the predominant isoform. Sequential (Z) scanning through *en face* vessel preparations suggested COX-2 was more abundant in the endothelial and intraplaque layers than the media. Images are merges of red (COX1 or COX2), green (CD31 – endothelial cells) and blue (DAPI – cell nuclei) channels. n = 4–6.

It is generally accepted that the inflammation associated with atherosclerosis leads to the induction of COX-2 [18], [31], [32], [33], [34]. However, COX-1 remains detectable in atherosclerotic tissue and the relative contribution of COX-2 to prostacyclin production in atherosclerotic vessels remains unclear [38] [39]. This is clearly a critical question since the prevailing dogma dictates that, in patients who are likely to have pre-existing atherosclerosis, COX-2 drives local vascular prostacyclin production [13], an idea that has been adopted by the American Heart Association and has influenced prescribing advice [46]. We found evidence of some COX-2-like immunoreactivity in atherosclerotic vessels alongside abundant COX-1-like imunoreacitivty (Figure 2). This COX-2 staining was specific since it was lost in COX-2-deficient mouse vessels (Figure S2). Sequential (Z) scanning through vessels suggested COX-2-like immnuoreactivity was most abundant in the endothelial and intraplaque layers and somewhat less present in the media (Figure 2; Figure S2), although the heterogeneous composition and uneven morphology of the diseased vessels complicates precise localisation. Further, immunohistochemical analysis does not allow quantitative comparison of different protein levels, and protein levels, in turn, may poorly reflect bioactivity. Consequently, in this study we have systematically assessed the ability of isolated atherosclerotic vessels from apoE^−/−^ mice to release prostacyclin (measured as its stable breakdown product, 6-keto-PGF1α) and PGE_2_, and how this is altered by deletion of the COX-2 gene. In order to ensure that COX expression rather than the level of phospholipase A_2_ activation was the rate-limiting step in prostaglandin production we measured release after stimulation with Ca^2+^ ionophore A23187. Our analysis determined the role of COX-2 in prostacyclin release from segments of the brachiocephalic artery (Figure 2a), aortic arch (Figure 2b) and thoracic aorta, which were each heavily burdened with atherosclerosis (Figure 2c), as well as plaque-free areas of the thoracic aorta (Figure 2d).

Previous studies have provided mixed results as to how total prostacyclin formation is altered in animal models of atherosclerosis [35], [36], [37]. Here we observed similar levels of prostacyclin release between vessels burdened with atherosclerosis and those free of disease by gross examination (Figure 2c,d). Moreover, regardless of the level of atherosclerosis, COX-2 did not detectably contribute to prostacyclin production by these isolated vessels (Figure 2). Similar results were seen when PGE_2_ release by the same vessels was measured (Figure 2). The physical trauma of removal of the vessel from the animal, *ex vivo* assay conditions and A23187 stimulation result in maximum activation of the vessel, conditions which may not reflect those *in vivo* and which may confound interpretation of these results. In order to address this we also measured plasma levels of 6-keto-PGF1α, a marker we have previously found to reflect systemic vascular prostacyclin production [24]. 6-keto-PGF1α was readily detectable in plasma and the levels did not differ between apoE^−/−^/COX-2^+/+^ (419±106 pg/ml) and apoE^−/−^/COX-2^−/−^ mice (436±66 pg/ml; p = 0.90). These findings are consistent with previous reports from hyperlipidemic rabbits [38] and our previous observations in healthy mouse vessels where COX-2 deletion did not alter vascular prostacyclin release, unless vessels are treated with inflammatory or mitogenic stimuli to induce COX-2 [24] or plasma prostacyclin unless animals were treated with high dose of LPS [47]. Whereas COX-1 deletion removed prostacyclin release *in vitro* and *in vivo* by >90% [24], [30]. Taken together, our data demonstrate that, despite modest enzyme induction, COX-2 does not meaningfully drive local vascular prostacyclin production in diseased plaque burdened atherosclerotic vessels or, as in healthy animals [24], [30] contribute to global circulatory prostacyclin release in animals with atherosclerosis.

**Figure 3:**
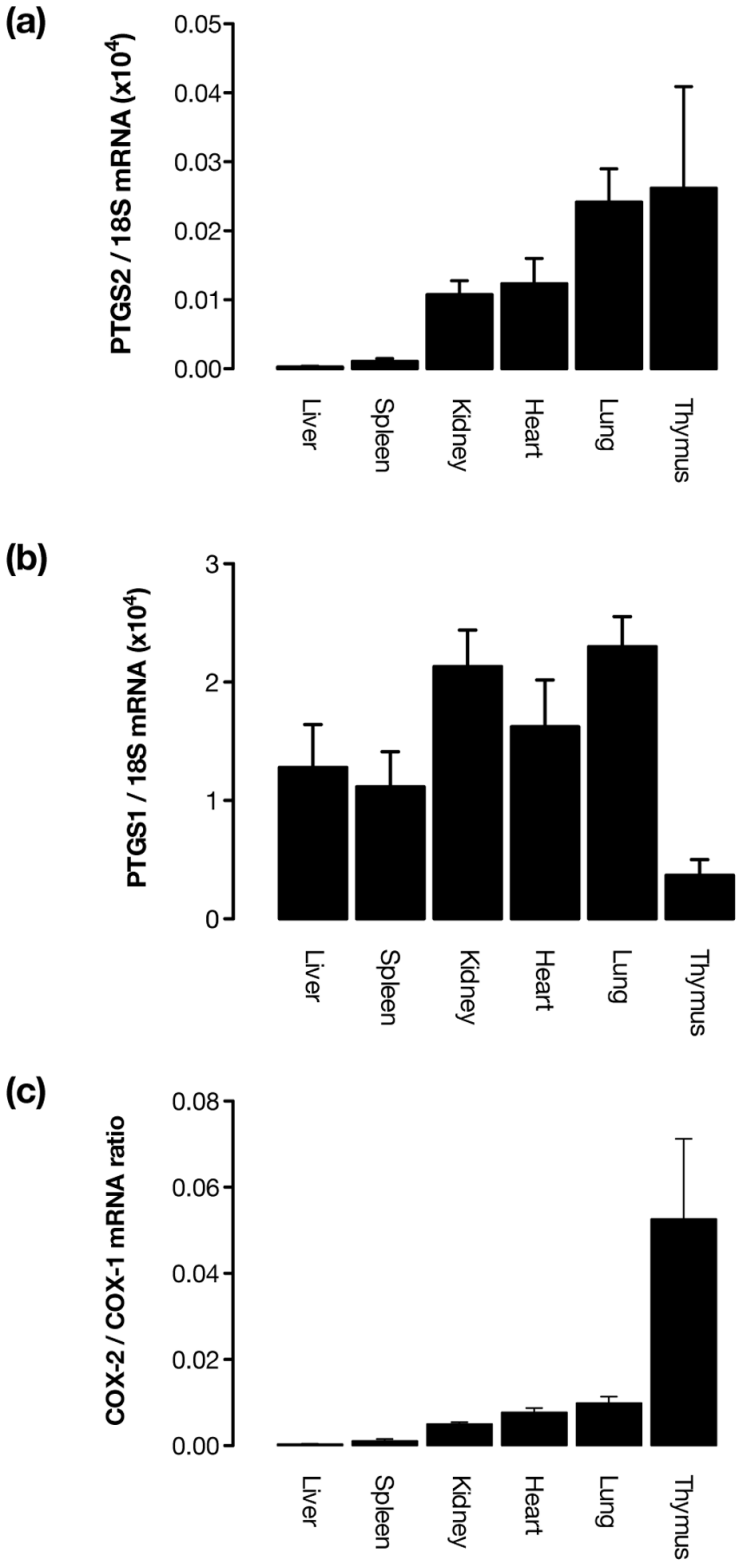
COX gene expression in tissue from atherosclerotic apoE^−/−^ mice. COX-2 (ptgs2) (a) and COX-1 (ptgs1) (b) gene expression levels measured by quantitative RT-PCR in a panel of tissues from atherosclerotic apoE^−/−^ mice indicated that COX-1 was expressed at a similar level and was the dominant isoform across all studied tissues. COX-2 expression showed more variation across tissues, with the highest levels present in the thymus, which also had the greatest COX-2:COX-1 expression ratio of any tissue examined (c). n = 4-6.

These observations however leave us now with no explanation of how COX-2 inhibition or gene deletion exacerbates atherosclerosis. Thus, with the idea that COX-2 mediates prostacyclin release in healthy [24] and atherosclerotic vessels (this study) removed, we must now address the possibility that COX-2 in sites remote to the affected vessel can protect indirectly. When doing so, it must be remembered that COX-2 may be expressed not only in parenchyma, but also in organ specialized microvasculature or other stromal cells. Indeed, endothelial specific COX-2 has been shown to protect against atherogenesis [39], despite our findings that vascular COX-2 has no role for prostacyclin production in affected vessels. In order to explore these hypotheses, we profiled COX-1 and COX-2 gene expression in a panel of tissues from atherosclerotic apoE^−/−^ mice to find sites where loss of COX-2 may influence pathophysiology. COX-2 message was differentially expressed across tissues with highest levels present in the thymus, and lowest in the liver (Figure 3a), results that are consistent with our recent observations using a COX-2 luciferase mouse revealing COX-2 gene hot spots in these tissues [30]. COX-1 expression was more consistent between tissues (Figure 3b) and in agreement with our previous observations of COX activity in tissues from healthy animals, was the dominant isoform expressed in all tissues examined. Interestingly, the thymus, the site of T-lymphocyte maturation, possessed not only the highest level of COX-2 expression, but also the lowest level of COX-1 expression, suggesting prostanoid production in this tissue may be relatively dependent on COX-2 in this model (Figure 3c).

### Effect of COX-2 deletion on the transcriptome in atherosclerotic mice; identification of T-lymphocyte pathways

**Figure 4:**
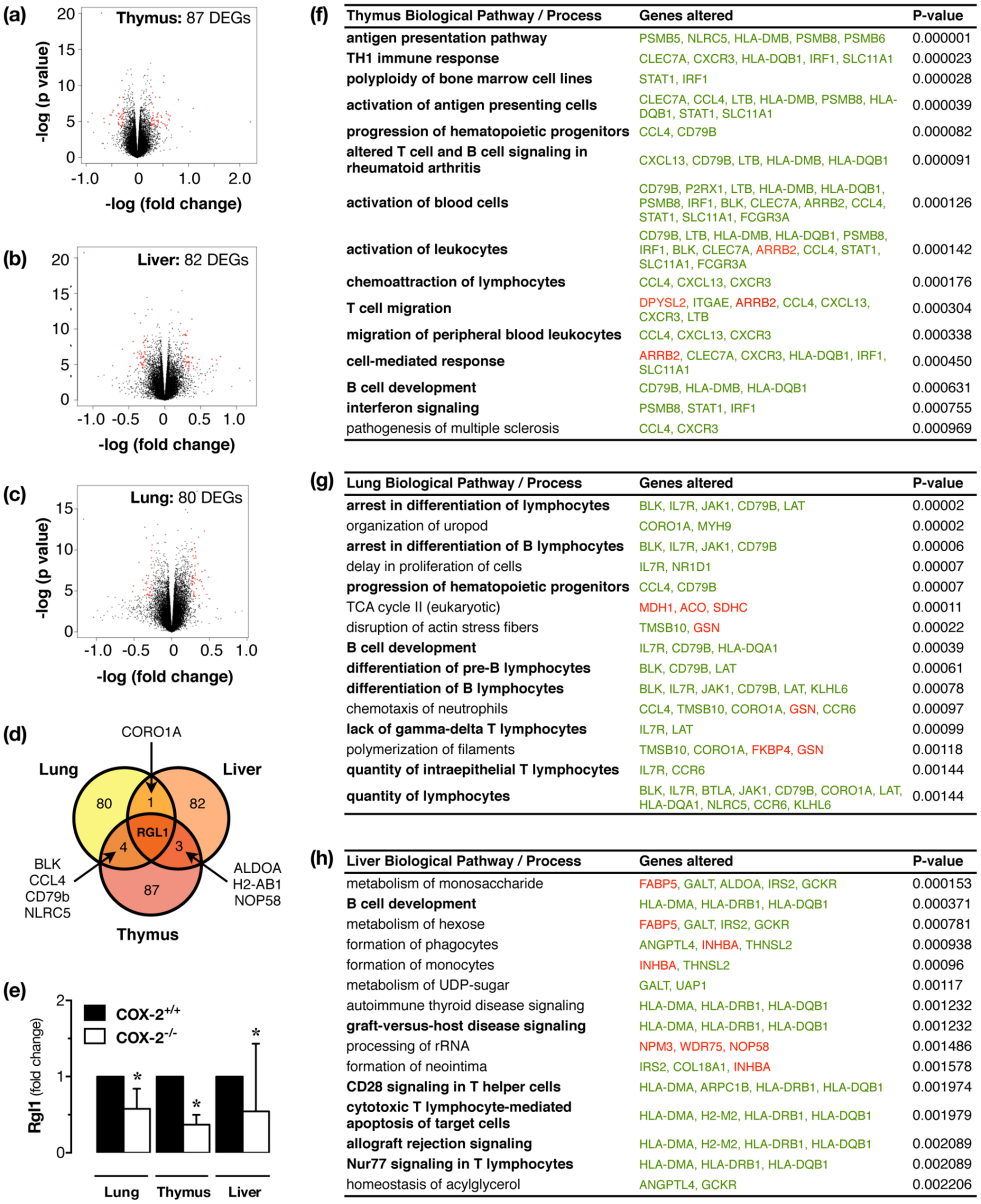
Deletion of COX-2 is associated with up-regulation of lymphocyte-related gene expression patterns, and down-regulation of rgl1 in tissue from fat-fed apoE^−/−^ mice. Microarray analysis indicated 82, 87 and 90 differentially expressed genes (DEGs), respectively, in thymus (a), liver (b) and lung (c) from fat-fed apoE^−/−^/COX-2^+/+^ and apoE^−/−^/COX-2^−/−^ mice. Volcano plot summaries of these data sets illustrate the distribution of effect size and statistical significance. Gene expression differing between genotypes >1.2-fold with p<0.05 highlighted in red, were considered for pathway analysis. Differential gene expression patterns demonstrated only weak overlap between tissues, but one gene, rgl1, was altered (down-regulated) in all tissues studied (d). Rgl1 down-regulation was confirmed in each tissue of apoE^−/−^/COX-2^−/−^ mice by qPCR (e). Ingenuity pathway analysis of this data showed that in the thymus (f), liver (g) and lung (h), biological pathways/processes related to T- and/or B-lymphocyte function were amongst the most consistently altered pathways in each tissue. Tables show the top 15 altered biological pathways/processes and implicated genes (green: increased expression; red: decreased expression). Statistical testing of microarray data was performed using a linear model for microarray data modified t-test. n = 4.

**Figure 5:**
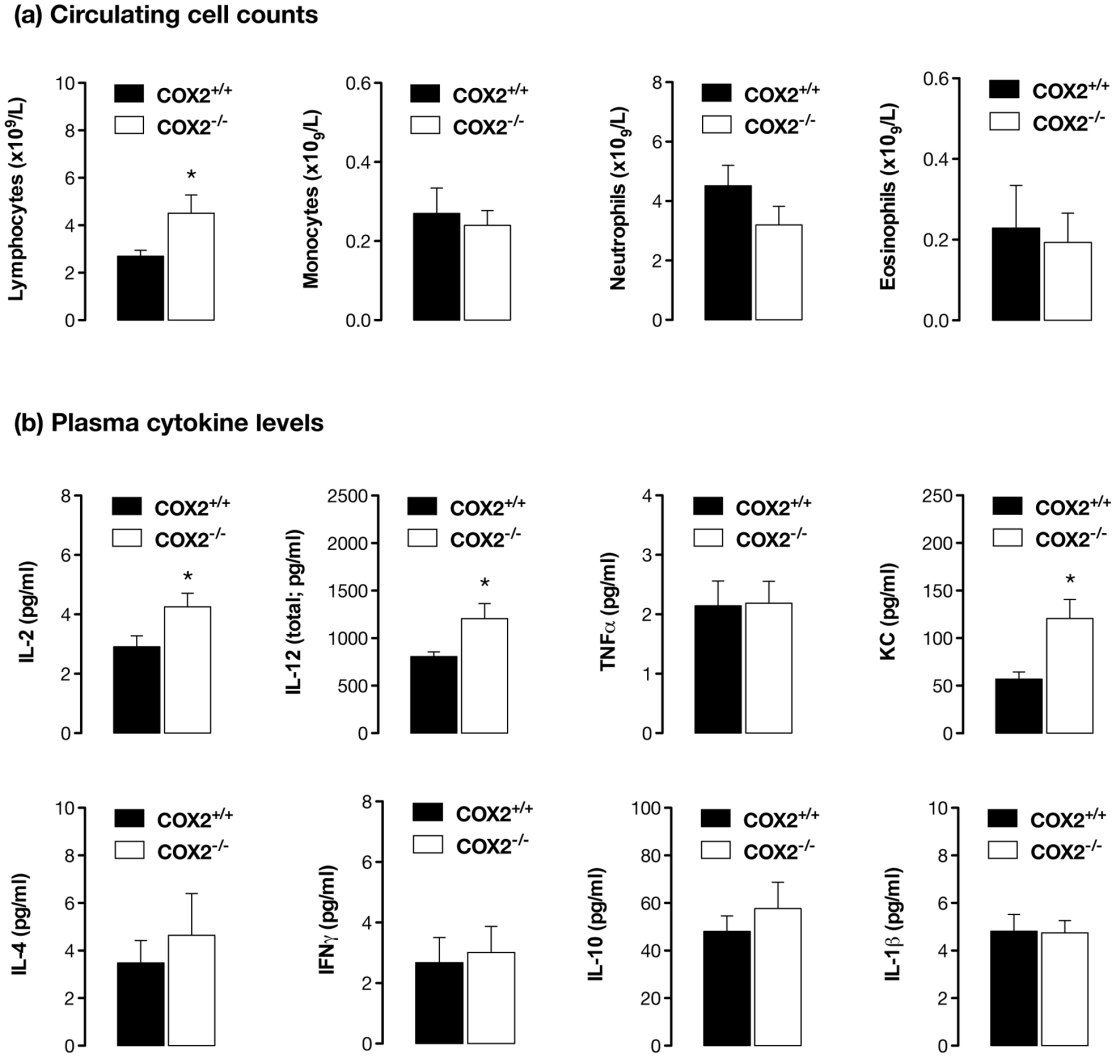
COX-2 deletion increases circulating levels of lymphocytes and lymphocyte-related cytokines in fat-fed apoE^−/−^ mice. Measurement of plasma levels of cytokines by multiplex immunoassay (a) demonstrated an increased in the lymphocyte-related cytokines IL-2 and IL-12 (total) and the neutrophil chemoattractant KC, but no change in levels of TNFα, IL-1β, IL-4, IL-10 or IFNγ. Cell counts in whole blood (b), measured in parallel, indicated an increase in circulating lymphocytes, but not monocytes, neutrophils or eosinophils. *; p<0.05 vs apoE^−/−^/COX-2^+/+^ by unpaired t-test; n = 10–25.

**Figure 6:**
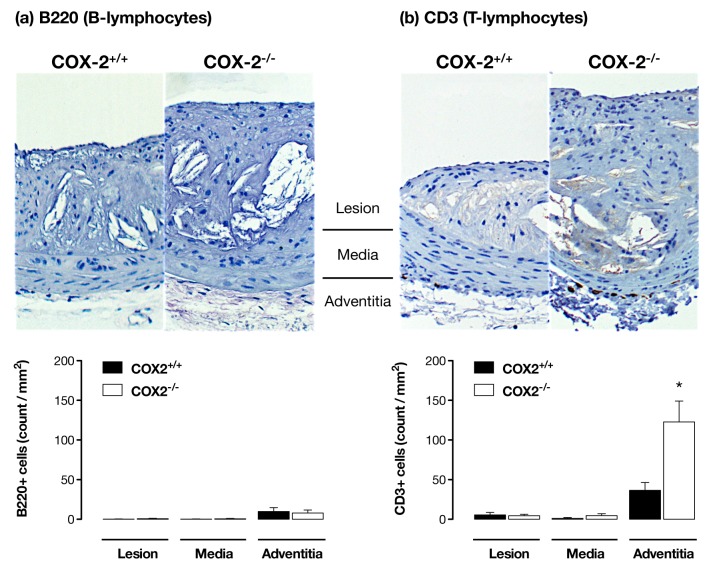
COX-2 deletion enhances T-lymphocyte infiltration into the adventitia of atherosclerotic vessels. Atherosclerotic lesions in the brachiocephalic artery of fat-fed apoE^−/−^/COX-2^+/+^ and apoE^−/−^/COX-2^−/−^ mice were examined for B- and T-lymphocytes respectively by immunohistochemistry for B220 (a) and CD3 (b). B220+ cells were rarely observed in vessels of either genotype. By contrast, CD3+ cells were present as focal accumulations in the adventitial layer of apoE^−/−^ mice, and the number and density of CD3+ cells present here was increased by deletion of COX-2. *; p<0.05 vs apoE^−/−^/COX-2^+/+^ by unpaired t-test; n = 5–10.

To generate new ideas about how COX-2 at distant sites might regulate atherosclerotic disease in the vessel wall we took a systems biology approach and measured the transcriptome of tissues from apoE^−/−^/COX-2^+/+^ and apoE^−/−^/COX-2^−/−^ mice. We examined the thymus, the highest COX-2 expressing tissue found, the lung, which is highly vascularised and sensitive to inflammation, and the liver, the major site of lipid metabolism. We then performed pathway analysis of microarray data to understand how changes in gene expression might influence biological processes (Figure 4, Figures S3-S5). In all three tissues, gene pathways involved in lymphocyte function and antigen presentation were amongst those most consistently and significantly altered (Figure 4, Figures S3-S5). To validate the importance of these pathways, we measured inflammatory cell counts in blood and found increased numbers of circulating lymphocytes in atherosclerotic mice lacking COX-2, but normal levels of monocytes, neutrophils, and eosinophils (Figure 5a). Similarly, deletion of COX-2 was associated with an increase in plasma levels of the lymphocyte cytokines IL-2 and IL-12, as well as the neutrophil chemoattractant, KC (Figure 5b). To determine if these changes extended to the atherosclerotic vessels themselves we examined T- (CD3^+^) and B-lymphocyte (B220^+^) accumulation in histological sections. B-lymphocytes were rarely observed in atherosclerotic vessels (Figure 6a), but T-lymphocytes were present as focal accumulations in the adventitial layer (Figure 6b). Crucially, the number of T-lymphocytes present was increased in atherosclerotic vessels from apoE^−/−^ lacking COX-2 (Figure 6b).

These findings suggest that T-lymphocytes may mediate enhanced atherosclerosis in COX-2-deficient apoE^−/−^ mice. Mature T-lymphocytes themselves, however, do not normally express COX-2 [48], [49], [50], and indeed, it has been reported that T-lymphocyte-specific COX-2-deletion does not alter atherosclerosis in an apoE-deficient model [50]. By contrast, it is known that deletion or inhibition of COX-2 alters the development of T-lymphocytes in the thymus [51], a site we have identified as highly expressing COX-2 in these mice. Further, prostanoids are reported to regulate a range of T-lymphocyte functions including proliferation, apoptosis and Th1/Th2 balance [48]. This latter effect may be particularly relevant to our study because we observed that deletion of COX-2 produced an increase in pro-atherogenic Th1-type cytokines (IL-2 and IL-12) and genes (STAT1, IRF1) but not Th2-type cytokines (IL-4, IL-10). A full gene validation and interpretation of how the thymus and associated lymphocyte pathways may be linked to COX-2, NSAIDs and atherosclerosis is beyond the scope of this study and remains the subject of investigation. Nevertheless, these ‘proof of concept’ studies provide a direction by which the cardioprotective properties of COX-2 at sites distant to the vessel may be identified.

### Association between COX-2 and the novel target gene rgl1 in tissue from atherosclerotic mice

In addition to lymphocyte-related pathways, our microarray analysis of tissue from apoE^−/−^ mice indicated that COX-2 was highly associated with a novel target gene, ral guanine nucleotide dissociation stimulator-like 1 (rgl1). Indeed, rgl1 was amongst strongly altered (down-regulated) genes in all three examined tissues (thymus, lung, liver), and the only common altered gene between them (Figure 4d; Figures S3-S5). This was confirmed in each tissue by qPCR (Figure 4e). The mechanism of rgl1 down-regulation is unclear since it was observed in tissues with both high (thymus, lung) and low (liver) COX-2 expression. This suggests the link between rgl1 and COX-2 must be mediated either (a) by a single COX-2 expressing cell type present in all tissues such as leucocytes or nerves, or (b) by a circulating factor or other systemic influence triggered by loss of COX-2 at a distant site.

There is little literature describing the function and regulation of rgl1 in mammalian systems. Rgl1 is a Ral guanine nucleotide exchange factor (RalGEF) closely related to RalGDS (Ral guanine nucleotide dissociation stimulator). RalGEFs are activated by Ras (and related molecules) and catalyse the activation of small G-proteins of the Ral family. The Ras/RalGEF/Ral pathway has been linked to many biological effects (reviewed in [52]) including proliferation, migration and inflammation (NFκB activation). If and how down-regulation of rgl1 by COX-2 deletion might regulate atherogenesis is unclear since reduced Ral signaling might be expected to *reduce* inflammation and proliferation. It should also be noted that Ras/RalGEF/Ral is a key pathway in cancer [52] and as such our findings may be of equal or greater relevance to understanding how NSAIDs exert their chemo-preventative effects. Clearly the mechanism(s) by which COX-2 regulates rgl1 expression and the biological consequences of this warrant further investigation.

### Summary and conclusions

Our previous work showed that COX-1, and not COX-2, mediates prostacyclin release in healthy vessels, and that urinary metabolites of prostacyclin do not reflect those in the circulation [24]. Here, we extend these observations, making the critical observation that COX-2 protects against atherosclerosis, but that this protection is mediated at some distant site and independent of local prostacyclin by the vessel. With the idea that COX-2 mediates vascular prostacyclin release removed, we must now revisit the question of where COX-2 is expressed and how this might influence vascular disease at distant sites. In this study we have performed microarray analysis of gene expression and used a systems biology approach to identify lymphocyte pathway signatures. This leads us to propose a role for the thymus and T-lymphocytes in the increased atherosclerosis observed in mice lacking COX-2. In addition, we have identified a novel target gene, rgl1, which is strongly linked to COX-2 in tissue from atherosclerotic mice.

The importance of this study is to show that in a model where COX-2 protects against atherosclerosis, this is independent of prostacyclin at the site of vascular lesions. Our study does not provide definitive answers but offers important new hypotheses for investigation and illustrates approaches that will, in the future, lead to a full understanding of how COX-2 might protect the cardiovascular system. NSAIDs remain the most common over the counter medications taken worldwide and there is a clear requirement for the continued development of new drugs in this class. These should spare the cardiovascular system as well as the gut, to fulfill their potential as anti-inflammatory drugs as well as in new clinical indications such as cancer.

## Supporting Information

Figure S1 **COX-2 deletion increases plaque macrophage but not lipid and smooth muscle content.** Atherosclerotic lesions in the brachiocephalic artery of fat-fed apoE^−/−^/COX-2^+/+^ and apoE^−/−^/COX-2^−/−^ mice were examined for estimated lipid content, smooth muscle/ myofibroblast content and macrophage content respectively by measuring intra-plaque voids in elastic Van Gieson (EVG) stained histological sections (a), or by immunohistochemistry for α-smooth muscle actin (αSMA; b) and Mac2 (c). Intra-plaque voids reminiscent of extracellular cholesterol crystals and Mac2 immunoreactivity were abundant in the intimal layer of all student vessels but rarer in the medial and adventital layers, whereas αSMA was present in both the intimal and medial layers. The relative abundance of lipid-like voids and αSMA immunoreactivity was not altered by COX-2 deletion. By contrast, vessels from apoE^−/−^ mice lacking COX-2 exhibited significantly more Mac2 immunoreactivity than vessels from control mice. *; p<0.05 vs apoE^−/−^/COX-2^+/+^ by unpaired t-test; n = 8–10.
(PDF)

Figure S2 **COX-2 is expressed in the atherosclerotic vessel and absent in COX-2^−/−^ tissue.** Atherosclerotic vessels from the lesser curvature of the aortic arch exhibited some COX-2-like immunoreactivity when examined by *en face* confocal immunofluorescence imaging. Sequential (Z) scanning through the vessel suggested this was present primarily in the endothelial and intraplaque layers with less present in the medial layers. In each case this was lost in COX-2-deficient mice demonstrating the antibody specificity. *; p<0.05 by two-way ANOVA. n = 4–6.
(PDF)

Figure S3 **Effect of COX-2 deletion on the transcriptome of the thymus in apoE^−/−^ mice.** Whole thymus from fat-fed apoE^−/−^/COX-2^+/+^ and apoE^−/−^/COX-2^−/−^ mice was examined for differential gene expression by microarray analysis. Genes exhibiting >1.25-fold expression level between genotypes are displayed. Data was analysed by the linear models for microarray analysis method. n = 4.
(PDF)

Figure S4 **Effect of COX-2 deletion on the transcriptome of the lung in apoE^−/−^ mice.** Whole lung from fat-fed apoE^−/−^/COX-2^+/+^ and apoE^−/−^/COX-2^−/−^ mice was examined for differential gene expression by microarray analysis. Genes exhibiting >1.25-fold expression level between genotypes are displayed. Data was analysed by the linear models for microarray analysis method. n = 4.
(PDF)

Figure S5 **Effect of COX-2 deletion on the transcriptome of the liver in apoE^−/−^ mice.** Whole liver from fat-fed apoE^−/−^/COX-2^+/+^ and apoE^−/−^/COX-2^−/−^ mice was examined for differential gene expression by microarray analysis. Genes exhibiting >1.25-fold expression level between genotypes are displayed. Data was analysed by the linear models for microarray analysis method. n = 4.
(PDF)
