# Supplementary material for: COX-2 Protects against Atherosclerosis Independently of Local Vascular Prostacyclin: Identification of COX-2 Associated Pathways Implicate Rgl1 and Lymphocyte Networks
Source: PLoS One. 2014 Jun 2;9(6):e98165. doi: 10.1371/journal.pone.0098165 (PMC4041570; doi:10.1371/journal.pone.0098165)
Supplement: Figure S1 — COX-2 deletion increases plaque macrophage but not lipid and smooth muscle content. Atherosclerotic lesions in the brachiocephalic artery of fat-fed apoE−/−/COX-2+/+ and apoE−/−/COX-2−/− mice were examined for estimated lipid content, smooth muscle/ myofibroblast content and macrophage content respectively by measuring intra-plaque voids in elastic Van Gieson (EVG) stained histological sections (a), or by immunohistochemistry for α-smooth muscle actin (αSMA; b) and Mac2 (c). Intra-plaque voids reminiscent of extracellular cholesterol crystals and Mac2 immunoreactivity were abundant in the intimal layer of all student vessels but rarer in the medial and adventital layers, whereas αSMA was present in both the intimal and medial layers. The relative abundance of lipid-like voids and αSMA immunoreactivity was not altered by COX-2 deletion. By contrast, vessels from apoE−/− mice lacking COX-2 exhibited significantly more Mac2 immunoreactivity than vessels from control mice. *; p<0.05 vs apoE−/−/COX-2+/+ by unpaired t-test; n = 8–10. (PDF) [file pone.0098165.s001.pdf]

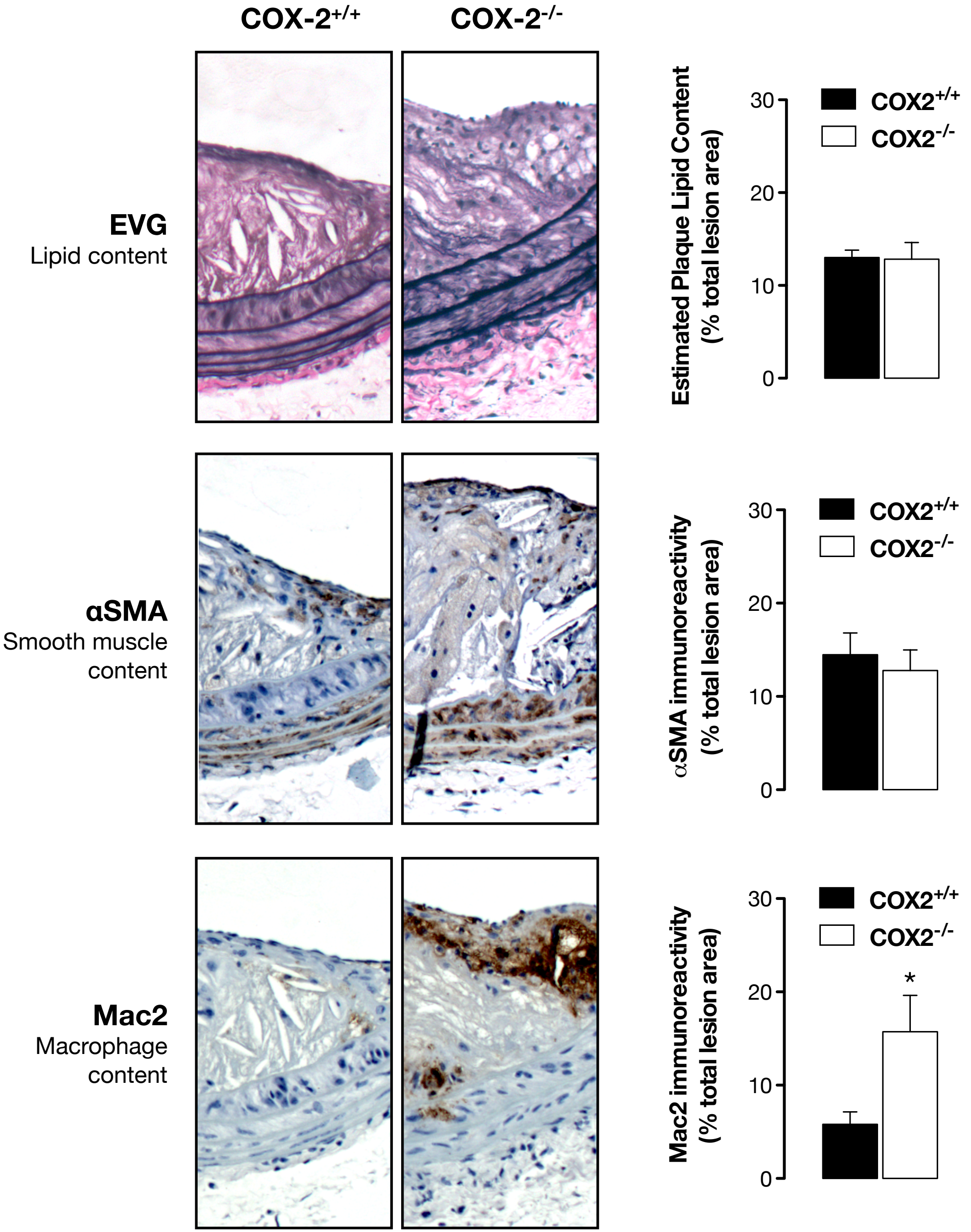

COX-2+/+
COX-2-/-
EVG
Lipid content
αSMA
Smooth muscle content
Mac2
Macrophage content
Estimated Plaque Lipid Content (% total lesion area)
αSMA immunoreactivity (% total lesion area)
Mac2 immunoreactivity (% total lesion area)
COX2+/+
COX2-/-
0
10
20
30
*
