# Supplementary material for: COX-2 Protects against Atherosclerosis Independently of Local Vascular Prostacyclin: Identification of COX-2 Associated Pathways Implicate Rgl1 and Lymphocyte Networks
Source: PLoS One. 2014 Jun 2;9(6):e98165. doi: 10.1371/journal.pone.0098165 (PMC4041570; doi:10.1371/journal.pone.0098165)
Supplement: Figure S2 — COX-2 is expressed in the atherosclerotic vessel and absent in COX-2−/− tissue. Atherosclerotic vessels from the lesser curvature of the aortic arch exhibited some COX-2-like immunoreactivity when examined by en face confocal immunofluorescence imaging. Sequential (Z) scanning through the vessel suggested this was present primarily in the endothelial and intraplaque layers with less present in the medial layers. In each case this was lost in COX-2-deficient mice demonstrating the antibody specificity. *; p<0.05 by two-way ANOVA. n = 4–6. (PDF) [file pone.0098165.s002.pdf]

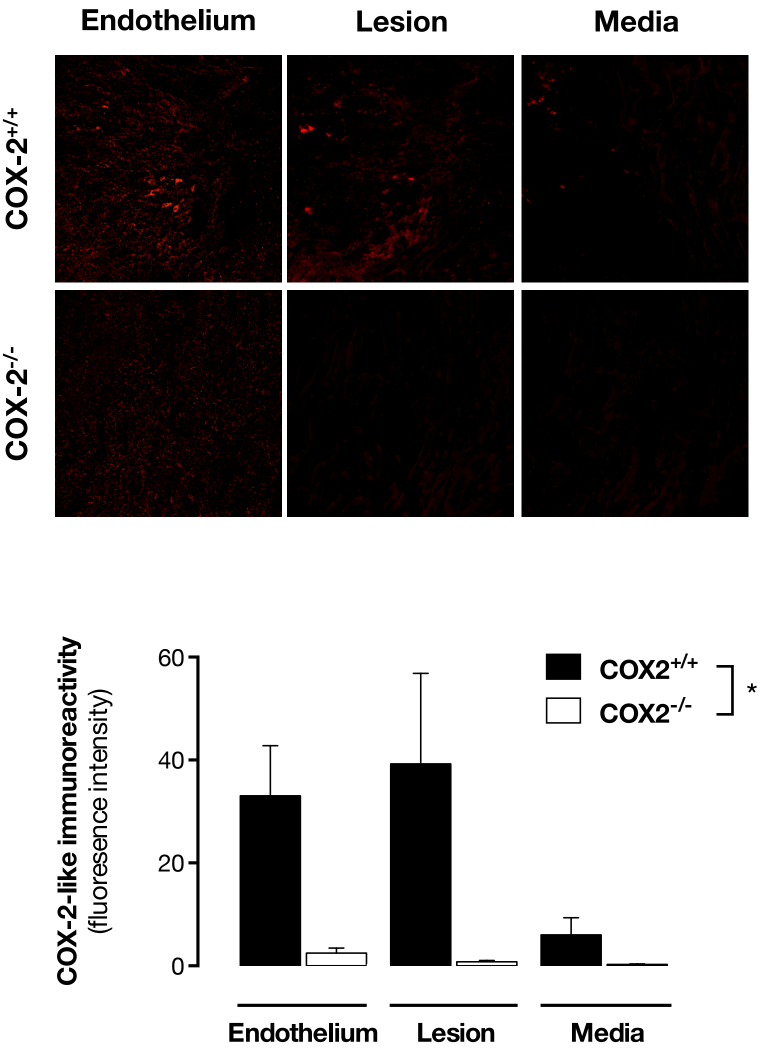

Endothelium
Lesion
Media
COX-2+/+
COX-2-/-
COX-2-like immunoreactivity
(fluoresence intensity)
60
40
20
0
COX2+/+
COX2-/-
*
Endothelium
Lesion
Media
