# Supplementary material for: COX-2 Protects against Atherosclerosis Independently of Local Vascular Prostacyclin: Identification of COX-2 Associated Pathways Implicate Rgl1 and Lymphocyte Networks
Source: PLoS One. 2014 Jun 2;9(6):e98165. doi: 10.1371/journal.pone.0098165 (PMC4041570; doi:10.1371/journal.pone.0098165)
Supplement: Figure S3 — Effect of COX-2 deletion on the transcriptome of the thymus in apoE−/− mice. Whole thymus from fat-fed apoE−/−/COX-2+/+ and apoE−/−/COX-2−/− mice was examined for differential gene expression by microarray analysis. Genes exhibiting >1.25-fold expression level between genotypes are displayed. Data was analysed by the linear models for microarray analysis method. n = 4. (PDF) [file pone.0098165.s003.pdf]

Symbol	Name	Fold Change	P-value
PSMB6	proteasome (prosome, macropain) subunit, beta type, 6	4.61	0.03224
MZB1	marginal zone B and B1 cell-specific protein	2.13	0.00890
GPS2	G protein pathway suppressor 2	1.74	0.00385
P2RX1	purinergic receptor P2X, ligand-gated ion channel, 1	1.56	0.01826
ITGAE	integrin, alpha E	1.54	0.02464
FCGR3A	Fc fragment of IgG, low affinity IIIa, receptor (CD16a)	1.52	0.01654
FCRLA	Fc receptor-like A	1.49	0.04370
GNB4	guanine nucleotide binding protein, beta polypeptide 4	1.48	0.01462
Irgm2	immunity-related GTPase family M member 2	1.48	0.01193
HBB	hemoglobin, beta	1.45	0.04030
TXNDC5	thioredoxin domain containing 5 (endoplasmic reticulum)	1.43	0.00039
Igtp	interferon gamma induced GTPase	1.43	0.00072
NLRC5	NLR family, CARD domain containing 5	1.41	0.03802
STAT1	signal transducer and activator of transcription 1, 91kDa	1.39	0.02091
CXCL13	chemokine (C-X-C motif) ligand 13	1.38	0.01808
CD79B	CD79b molecule, immunoglobulin-associated beta	1.38	0.03354
IRF1	interferon regulatory factor 1	1.35	0.00306
TRIB2	tribbles homolog 2 (Drosophila)	1.33	0.03413
TSPAN32	tetraspanin 32	1.32	0.02771
TCTEX1D2	Tctex1 domain containing 2	1.32	0.01359
COX6A2	cytochrome c oxidase subunit VIa polypeptide 2	1.32	0.04085
HVCN1	hydrogen voltage-gated channel 1	1.32	0.03028
EDEM2	ER degradation enhancer, mannosidase alpha-like 2	1.32	0.02016
SAA2	serum amyloid A2	1.31	0.04901
PSMB5	proteasome (prosome, macropain) subunit, beta type, 5	1.31	0.01557
Cd59a	CD59a antigen	1.31	0.01609
CCL4	chemokine (C-C motif) ligand 4	1.30	0.02923
KLK3	kallikrein-related peptidase 3	1.28	0.04499
CXCR3	chemokine (C-X-C motif) receptor 3	1.28	0.04831
RAB32	RAB32, member RAS oncogene family	1.28	0.00297
SLC11A1	solute carrier family 11, member 1	1.26	0.04331
HLA-DMB	major histocompatibility complex, class II, DM beta	1.26	0.02731
FAM91A1	family with sequence similarity 91, member A1	-1.25	0.04638
DNMT1	DNA (cytosine-5-)-methyltransferase 1	-1.26	0.02010
FUS	fused in sarcoma	-1.27	0.02096
PSMC5	proteasome (prosome, macropain) 26S subunit, ATPase, 5	-1.27	0.00308
RBM47	RNA binding motif protein 47	-1.28	0.01508
LSM14B	LSM14B, SCD6 homolog B (S. cerevisiae)	-1.28	0.03968
TPM3	tropomyosin 3	-1.29	0.02526
BZW2	basic leucine zipper and W2 domains 2	-1.29	0.03806
CCNL1	cyclin L1	-1.30	0.03348
RANBP1	RAN binding protein 1	-1.30	0.00643
COX7A2L	cytochrome c oxidase subunit VIIa polypeptide 2 like	-1.30	0.01405
FAM168B	family with sequence similarity 168, member B	-1.31	0.00643
SNRNP70	small nuclear ribonucleoprotein 70kDa (U1)	-1.39	0.01050
ELAVL1	ELAV-like 1 (Hu antigen R)	-1.40	0.01254
RBM12	RNA binding motif protein 12	-1.42	0.01707
ARRB2	arrestin, beta 2	-1.45	0.01648
RPE	ribulose-5-phosphate-3-epimerase	-1.46	0.03965
CCRN4L	CCR4 carbon catabolite repression 4-like (S. cerevisiae)	-1.57	0.03424
SLC15A2	solute carrier family 15 (H+/peptide transporter), member 2	-1.57	0.02341
RGL1	ral guanine nucleotide dissociation stimulator-like 1	-1.63	0.00000
TRIM59	tripartite motif containing 59	-1.84	0.01416
LOC728392	uncharacterized LOC728392	-1.95	0.03239
