# Supplementary material for: COX-2 Protects against Atherosclerosis Independently of Local Vascular Prostacyclin: Identification of COX-2 Associated Pathways Implicate Rgl1 and Lymphocyte Networks
Source: PLoS One. 2014 Jun 2;9(6):e98165. doi: 10.1371/journal.pone.0098165 (PMC4041570; doi:10.1371/journal.pone.0098165)
Supplement: Figure S4 — Effect of COX-2 deletion on the transcriptome of the lung in apoE−/− mice. Whole lung from fat-fed apoE−/−/COX-2+/+ and apoE−/−/COX-2−/− mice was examined for differential gene expression by microarray analysis. Genes exhibiting >1.25-fold expression level between genotypes are displayed. Data was analysed by the linear models for microarray analysis method. n = 4. (PDF) [file pone.0098165.s004.pdf]

Symbol	Name	Fold Change	P-value
CD79B	CD79b molecule, immunoglobulin-associated beta	1.38	0.04185
SLC34A2	solute carrier family 34 (sodium phosphate), member 2	1.35	0.00020
Lyz1/Lyz2	lysozyme 2	1.34	0.02605
TBC1D10C	TBC1 domain family, member 10C	1.32	0.02027
MCF2L	MCF.2 cell line derived transforming sequence-like	1.32	0.00615
CCR6	chemokine (C-C motif) receptor 6	1.31	0.01405
HSD3B2	hydroxy-δ5-steroid dehydrogenase, 3β- and δ-isomerase 2	1.29	0.01323
NR1D1	nuclear receptor subfamily 1, group D, member 1	1.29	0.04498
SS18	synovial sarcoma translocation, chromosome 18	1.28	0.00033
Pisd-ps1	phosphatidylserine decarboxylase, pseudogene 1	1.27	0.00311
TMSB10	thymosin beta 4, X-linked	1.27	0.01172
C20orf160	chromosome 20 open reading frame 160	1.26	0.00001
CORO1A	coronin, actin binding protein, 1A	1.25	0.02550
TXN	thioredoxin	1.25	0.00195
BLK	B lymphoid tyrosine kinase	1.25	0.00162
ITM2B	integral membrane protein 2B	1.25	0.00114
GABARAPL1	GABA(A) receptor-associated protein like 1	-1.25	0.02322
PDLIM5	PDZ and LIM domain 5	-1.25	0.04087
DYNC1H1	dynein, cytoplasmic 1, heavy chain 1	-1.26	0.01708
TTYH3	tweety homolog 3 (Drosophila)	-1.26	0.04334
NDRG2	NDRG family member 2	-1.26	0.00036
ACO2	aconitase 2, mitochondrial	-1.26	0.01464
TP53INP2	tumor protein p53 inducible nuclear protein 2	-1.26	0.00839
SPTBN1	spectrin, beta, non-erythrocytic 1	-1.27	0.02902
TMEM66	transmembrane protein 66	-1.28	0.01629
VAMP3	vesicle-associated membrane protein 3 (cellubrevin)	-1.28	0.00014
ADH1C	alcohol dehydrogenase 1C (class I), gamma polypeptide	-1.28	0.04502
ART3	ADP-ribosyltransferase 3	-1.30	0.02807
FXYD3	FXYD domain containing ion transport regulator 3	-1.31	0.01230
SDHC	succinate dehydrogenase complex, subunit C	-1.35	0.00278
MYH6	myosin, heavy chain 6, cardiac muscle, alpha	-1.42	0.02029
GSN	gelsolin	-1.43	0.03303
RGL1	ral guanine nucleotide dissociation stimulator-like 1	-2.25	0.00007
