# Supplementary material for: COX-2 Protects against Atherosclerosis Independently of Local Vascular Prostacyclin: Identification of COX-2 Associated Pathways Implicate Rgl1 and Lymphocyte Networks
Source: PLoS One. 2014 Jun 2;9(6):e98165. doi: 10.1371/journal.pone.0098165 (PMC4041570; doi:10.1371/journal.pone.0098165)
Supplement: Figure S5 — Effect of COX-2 deletion on the transcriptome of the liver in apoE−/− mice. Whole liver from fat-fed apoE−/−/COX-2+/+ and apoE−/−/COX-2−/− mice was examined for differential gene expression by microarray analysis. Genes exhibiting >1.25-fold expression level between genotypes are displayed. Data was analysed by the linear models for microarray analysis method. n = 4. (PDF) [file pone.0098165.s005.pdf]

Symbol	Name	Fold Change	P-value
C10orf10	chromosome 10 open reading frame 10	2.06	0.04478
HYI	hydroxypyruvate isomerase (putative)	1.71	0.01443
ANGPTL4	angiopoietin-like 4	1.66	0.02986
AQP8	aquaporin 8	1.65	0.02021
HLA-DQB1	major histocompatibility complex, class II, DQ beta 1	1.59	0.03631
HLA-DRB1	major histocompatibility complex, class II, DR beta 1	1.54	0.04714
H2-M2	histocompatibility 2, M region locus 2	1.39	0.01892
SGK2	serum/glucocorticoid regulated kinase 2	1.37	0.02777
LPIN2	lipin 2	1.37	0.04146
Acat2/Acat3	acetyl-Coenzyme A acetyltransferase 2	1.32	0.04680
CEBPE	CCAAT/enhancer binding protein (C/EBP), epsilon	1.32	0.03659
GCKR	glucokinase (hexokinase 4) regulator	1.31	0.01647
TBC1D17	TBC1 domain family, member 17	1.31	0.01537
NAGK	N-acetylglucosamine kinase	1.30	0.00037
SH3BGRL3	SH3 domain binding glutamic acid-rich protein like 3	1.30	0.02647
DEDD2	death effector domain containing 2	1.27	0.02643
HLA-DMA	major histocompatibility complex, class II, DM alpha	1.26	0.02340
CORO1A	coronin, actin binding protein, 1A	1.26	0.04475
GSTM5	glutathione S-transferase mu 5	1.26	0.03310
TCEA3	transcription elongation factor A (SII), 3	1.26	0.03793
IRS2	insulin receptor substrate 2	1.26	0.01567
ALDOA	aldolase A, fructose-bisphosphate	1.25	0.02586
GSTM4	glutathione S-transferase mu 4	1.25	0.01405
CRAT	carnitine O-acetyltransferase	1.25	0.02380
CHORDC1	cysteine and histidine-rich domain (CHORD) containing 1	-1.25	0.01172
C5orf43	chromosome 5 open reading frame 43	-1.26	0.01411
ADAT2	adenosine deaminase, tRNA-specific 2	-1.26	0.02575
TXNL4A	thioredoxin-like 4A	-1.26	0.01699
CTPS1	CTP synthase 1	-1.26	0.02601
ETNK2	ethanolamine kinase 2	-1.27	0.00055
Npm3-ps1	nucleoplasmin 3, pseudogene 1	-1.27	0.00844
RNASE4	ribonuclease, RNase A family, 4	-1.28	0.01113
C21orf91	chromosome 21 open reading frame 91	-1.29	0.03191
MSH6	mutS homolog 6 (E. coli)	-1.30	0.00938
ITIH4	inter-alpha-trypsin inhibitor heavy chain family, member 4	-1.30	0.04577
FABP5	fatty acid binding protein 5 (psoriasis-associated)	-1.31	0.01658
GDF10	growth differentiation factor 10	-1.33	0.00022
FOXA3	forkhead box A3	-1.35	0.01045
NOP58	NOP58 ribonucleoprotein homolog (yeast)	-1.36	0.00332
DYNLL1	dynein, light chain, LC8-type 1	-1.40	0.03771
HSPH1	heat shock 105kDa/110kDa protein 1	-1.51	0.00701
MCM6	minichromosome maintenance complex component 6	-1.65	0.03540
RGL1	ral guanine nucleotide dissociation stimulator-like 1	-2.18	0.00000
